# The relationship between severity of apraxia of speech and working memory

**DOI:** 10.1590/S1980-57642010DN40100011

**Published:** 2010

**Authors:** Karin Zazo Ortiz, Fernanda Chapchap Martins

**Affiliations:** 1 PhD in Neuroscience from UNIFESP, Professor at the Department of Speech Pathology and Audiology, Department of Speech Pathology and Audiology, UNIFESP, São Paulo SP, Brazil.; 2 Masters in Human Communication Disorders, Department of Speech Pathology and Audiology, UNIFESP, São Paulo SP, Brazil.

**Keywords:** apraxia, memory, articulation disorders

## Abstract

Based on previously observed relationships between working memory (WM) and speech, the current study investigated the relationship between degree of oral apraxia (AOS) and WM capacity.

**Methods:**

This study involved assessment and classification of degree of apraxia of speech in 22 apraxic participants and evaluation of WM capacity using digit span and word-list repetition tests. Both tests were able to assess the phonoarticulatory loop, while the Rey Auditory Verbal Learning Test investigated the phonoarticulatory loop and the episodic buffer.

**Results:**

Independently from the degree of apraxia of speech, all of participants presented compromise in WM.

**Conclusions:**

The data presented might suggest that individuals with AOS typically have WM impairment, but it is still not clear if the severity of AOS is related to WM capacity. Future studies could verify the relationship between the severity of apraxia and the severity of WM deficits.

## Abstract (pt)

Considerando-se que estudos anteriores observaram correlações entre a memória operacional e a apraxia de fala, o objetivo do presente estudo foi o de verificar se há correlação entre a gravidade da apraxia de fala e a alteração da memória operacional.

**Métodos:**

Foram avaliados 22 pacientes apráxicos. Todos os participantes foram submetidos à avaliação da apraxia de fala. Para investigar a memória operacional, foram aplicados o teste de span de dígitos na ordem direta e inversa, um teste de repetição de palavras longas e curtas e o Rey Auditory Verbal Learning Test, que investiga, além da alça articulatória, o buffer episódico.

**Resultados:**

Independentemente do grau da apraxia de fala, todos os participantes tiveram comprometimento da memória operacional. No entanto, não foi observada correlação entre o grau da apraxia de fala e a presença de comprometimento da memória operacional.

**Conclusões:**

Os dados sugerem que indivíduos com apraxia de fala apresentam um déficit na memória operacional. No entanto, novos estudos devem investigar a correlação entre diferentes gravidades de apraxia de fala e diferentes graus de comprometimento da memória operacional.

The relationship between WM and motor planning of speech has been reported in previous studies.^1,2^ Determining which components are closely related to WM could assist and guide diagnosis and rehabilitation of apraxic patients. Therefore, it was important to investigate the involvement of both articulatory process and phonological storage in motor planning speech.

In 2000, Baddeley^3^ made the final additions to the WM model which currently encompasses the central executive, visuo-spatial sketchpad and phonoarticulatory loops, and episodic buffer. The central executive is responsible for the following functions: coordination of two activities performed simultaneously, strategic alternation during retrieval of memorized material, and selective servicing of stimuli and inhibition of other distracting stimuli.^4^ The visuo-spatial sketchpad acts as a system involved in generalizing and manipulating visual and spatial information. Burgess and Hitch^5^ have emphasized the importance of interaction between short and long term memories. Following discussion of results in the literature on these themes, these authors confirmed an important link between the two memory types. Although immediate repetition of a sequence is associated with the phonological loop, it is also influenced by long-term memory. Better performance in repeating familiar words than both non-familiar and non words exemplifies the connection between long-term memory and the phonological loop. These authors concluded that the models of short and long term memory ought to fit into a common network. Besides, when material to be memorized is phonemically similar, individuals encounter greater difficulties and exhibit shorter span. This occurs since similar items present less distinguishable cues than differing items, and are thus more prone to be forgotten.^6,7^ However, similarities in meaning do not show the same effect, suggesting that this subsystem is not responsible for semantic coding.^6^ With regard to word length, shorter words are easier to memorize and retrieve.

Gathercole and Baddeley^8^ attempted to link the role of the phonoarticulatory loop of the WM with the complex process of producing speech. The authors also linked these findings with other studies carried out, demonstrating that subvocal retention of the material in the articulatory loop involves processes used in the planning, and not the execution, of the oral emission.

Previous studies^1,2^ have verified that apraxics present reduced WM capacity suggestive of phonoarticulatory loop dysfunction and the results were interpreted as suggesting that apraxic individuals, who presented a disorder in motor planning of speech, failed in the subvocal rehearsal process and therefore presented a working memory deficit.

The present study aimed to verify the relationship between degree of apraxia and WM capacity.

## Methods

This study was carried out within the Speech Therapy Department of the São Paulo Federal University (UNIFESP), Brazil. A total of 20 patients were studied. These patients were selected according to the following inclusion criteria: brain lesion in the left hemisphere, presence of apraxia of speech, aged between 31 to 65 years. Of the participants included, 12 were male and 8 female. Schooling ranged from 1 to 15 years of education. All patients presented apraxia of speech associated to aphasia. Patients with fluent, severe or anomic aphasia were excluded from this study. The sites of lesions were confirmed through a neurological assessment and according to imaging exams. Two subjects presented brain lesion in the frontal region, 3 in the temporal region, 2 in the parietal region, 3 in the fronto-temporal, 5 parietal-temporal region, and 5 presented lesions in the parietal fronto-temporal region.

Apraxia of speech was diagnosed through assessment by a speech pathologist and a specific protocol^9^ was employed. Regarding the characterization of the apraxic disorder of participants, the applied protocol was able to classify these in terms of degree of the disorder: 50% presented mild apraxia of speech, 45.5% moderate, and 4.5% severe. This classification was only possible by means of thorough analysis of the following apraxic errors: greater difficulty in producing movements or speech when prompted, i.e. voluntarily, than when automatic;^10-12^ errors which are unpredictable and inconsistent; higher probability of errors on longer words or those having a greater grammatical load within a sentence,^10,11,13^ phoneme substitution errors;^10,11,14^ perseverations, antecipations, transpositions, additions or inclusions of phonemes;^10,11,14^ prosody deficit^10,13-15^ where errors intensify according to the complexity of the motor adjustment required to produce a given phoneme.^10^ All these manifestations were analyzed quantitatively and qualitatively in order to achieve an accurate diagnosis.

Participants signed the Free and Informed Consent Term. The study was approved by the Research Ethics Committee of UNIFESP under CEP number 0382/04.

Participants in this investigation underwent tasks assessing oral comprehension, working memory and apraxia of speech.

The tests employed in this study, together with procedures adopted are outlined below.

### Assessment of oral comprehension

Oral comprehension was investigated through application of the oral comprehension section of the Boston Diagnostic Aphasia Examination,^16^ called Complex Ideational Material. All individuals presenting sufficient oral comprehension to perform the tests were included in the study.

### Assessment of apraxia of speech

Only patients underwent apraxia of speech investigation. This was performed to classify the apraxias by degree of severity.

The Martins and Ortiz protocol^9^ was employed to assess apraxia, in which the following tasks were applied: word and sentence repetition, spontaneous and automatic speech, along with reading words and sentences aloud, enabling apraxia of speech to be identified and classified. In the knowledge that greater phoneme complexity and word length lead to a higher number of errors being committed,^13,17^ the prompts making up this protocol included words with different numbers of syllables, and phrases and phonemes of varying complexity. The examiner recorded an accurate transcription of patients’ speech, considering only specific errors whilst excluding language errors such as agrammatisms, anomias and semantic paraphasias, along with other errors unrelated to the apraxic picture.

The repetition of words and sentences task entailed reading of stimuli to the patient, and subsequent repetition of these by the participant. The spontaneous speech test consisted of description of a figure from a given thematic card, whilst automatic speech involved counting the numbers 1 to 20 and months of the year.

The reading aloud of words and phrases was performed in the same manner as for repetition, except patients were instructed to read the stimuli. In response analyses, both quantitative and qualitative observation of errors committed was fundamental, since assessment of quantity along with error types committed allowed patient performance to be graded as mild, moderate or severe.

In terms of apraxia severity, ten participants presented mild verbal apraxia and ten moderate.

### Assessment of memory

This assessment comprised three different tests: the word list repetition, the digit span forward and backward, and the Rey Auditory Verbal Learning Test (RAVLT).

**Word list repetition:** This test consisted of two and three-syllable words, in order to verify how memory influenced processing of these prompts.^18^ The individual was instructed to repeat orally presented lists containing two to six words. Items were presented at one second intervals and the response was oral. Participants had to repeat the list in the correct order, and when failing twice on same-length words and lists the test was concluded. Individual span was determined according to the maximum number of correctly repeated words. This test requires strict serial recall, so it can provide a sensitive measure of phonological loop.

**Digit span forward:** According to Baddeley and Hitch,^19^ the DS tests require functioning of WM and more specifically, of the phonological loop.

**Digit span backward:** This task is deemed more complex than the forward DS, since the information must be processed more times prior to being retrieved, thus placing greater demands on the WM.^4,20^

**RAVLT:** This test consists of 15 words (list A) which were read aloud by the examiner (with a one-second interval per item) five consecutive times. Each presentation was followed by the participant orally repeating as many words as they could recall from memory.

Instructions were repeated before each trial in order to minimize forgetting of the task. Following completion of five trials, a second list containing another 15 words (List B) was read and subsequently repeated by the participant. Immediately after this distracter, the individual was instructed to spontaneously repeat the words recalled from List A, where this procedure was repeated after 20 minutes. This final recall is influenced by the episodic buffer of the WM, as the task calls for long-term memory. It is important to note that these final recalls are not preceded by repeat readings by the examiner. After delayed recall, the recognition test was applied. The 15 words from list A was pooled with a list of another 15 words which were either phonologically or semantically similar, or presented no similarity to the lists given initially. The examiner read this list of 30 items and the patient was instructed to identify which items belonged to the original list and which were “new”. As responses were limited to yes or no, and individuals use the phonoarticulatory retrieval component only to compare the given prompt against that stored.

Regarding learning analyses, the method developed by Ivnik et al.^21^ in MOANS (*Mayo's Older Americans Normative Studies*) was employed, which standardizes scoring as follows:

**Total Learning (TL):** Total learning is established by summing up words recalled over the five trials.

**Learning Over Trials (LOT):** Calculated based on TL, adjusted for the first trial, that is, LOT=TL – (5 × number of words obtained in the first trial).

The RAVLT assesses a range of cognitive skills such as attention, different components of working memory and learning.^22-25^

Comparison between degree of apraxia and performance on memory tests were made using the independent *Student (t)* test.

A probability (*p*) of less than 0.05 was considered as statistically significant, except when a potential problem of multiple comparisons was identified. In this event, Bonferroni’s correction was employed. All tests were two-tailed. A ninety five per cent Confidence Interval (CI) was calculated for differences amongst means. All analyses were carried out using SPSS (*Statistical Package for the Social Sciences*) 11.5.1 for Windows.

## Results

Patients with apraxia of speech were subdivided into two sub groups: with mild and moderate apraxia, and their cognitive test scores compared, where no statistically significant difference was observed between them. Table 1 shows the performance of the 2 groups with mild and moderate/severe apraxia respectively, and presents a comparison of performance of the two subgroups.

**Table 1:** Comparison of individuals with mild or moderate/severe apraxia on cognitive tests.

	Mild M	SD	Mod/S M	SD	95% CI	T	DF	p
Comprehension test	7.5	2.2	6.6	0.8	-1.4 to 3.1	0.8	20	0.456
Short word span	2.6	0.7	2.6	1.0	-0.7 to 0.8	0.1	19	0.921
Long word span	2.5	0.5	2.7	-0.2	-0.8 to 0.5	-0.5	19	0.645
Digit span forward	3.2	0.8	3.4	1.4	-1.2 to 0.8	-0.4	20	0.702
Digit span backward	2.5	0.5	2.2	0.9	-0.4 to 0.9	0.9	20	0.385
RAVLT								
Immediate retrieval	3.6	1.4	3.5	3.7	-2.5 to 2.8	0.1	19	0.908
Delayed retrieval	4.3	2.2	4.0	3.3	-2.3 to 2.9	0.2	19	0.812
Learning	9.8	5.1	7.5	5.9	-2.7 to 7.4	1.0	19	0.343
Recognition	24.6	2.1	23.7	4.1	-2.1 to 3.9	0.6	19	0.550

Mod/S, moderate/severe apraxia; Mild, mild apraxia; M, mean; SD, standard deviation; CI, confidence interval; DF, degrees of freedom; p<0.05 after Bonferroni's correction;

* statistical significance.

## Discussion

Although the patients scored lower than was expected for normal subjects on oral comprehension subtest, there was no impact on performance of the other tests, as all participants enrolled on the study were able to follow the instructions. A number of hypotheses which may explain this discrepancy shall be addressed.

Firstly, the fact that all apraxics studied presented an associated aphasic disorder must be considered. Thus, this language difficulty is likely to have interfered in the results of the comprehension test in apraxic individuals. According to Darley,^10^ the occurrence of apraxia only is very rare. Even in expressive aphasia pictures, at least a mild disturbance in oral comprehension is expected in these cases.

Another justification, closely related to that outlined above, or of even greater significance, is the WM deficit found in all apraxics. The important role played by working memory in the oral and graphic process is well established in the literature. According to Baddeley,^4^ comprehension depends on the capacity of WM in some way. Moreover, Martin et al.^26^ reported a case of an individual with alterations in WM and difficulties understanding sentences.

The authors concluded that this comprehension disorder was due to difficulty in retaining semantic information in WM. In 1998, Engle and Conway^27^ also linked WM with comprehension. They reported that the phonoarticulatory loop is required when the sentence to be understood is long or highly complex. In addition to these situations, the WM influences comprehension of texts, oral or graphic when the ideas are not presented in a linear sequence or when pronouns are used to refer to previously cited names. In this study, the instructions were easy to follow.

### Relationship between degree of apraxia and WM capacity

In a previous study^2^ with the same apraxic patients, a statistically significant difference between the performance of apraxics and a control group, matched by age, sex and years of education, was noted across all memory tests: long and short word repetition and forward and reverse digit span.

Relationships between the degree of apraxia and performance obtained in memory trials were investigated in this study (Table 1). The results indicate that, independently of degree of the apraxia of speech, the deficit in WM is still observed, where this deficit does not increase with increased disturbance in speech. This data suggests that even when the disturbance in motor programming of speech is mild, it is able to compromise the subvocal mental rehearsal process and thus reduce the span of WM.

Initially considering only the results obtained on the digit span, which assesses attention and WM,^28^ the difference in performance between the forward and reverse order is notable. However, better performance on the digit span forward was expected as the processing needed by the WM is considered simpler than for the digit span backward which demands more complex processing. According to Wilde et al.,^20^ the digit span forward is performed primarily by the phonoarticulatory loop, whereas the digit span backward requires involvement of the central executive hence calling for greater participation of the WM. Unswoth and Engle^30^ stated that more complex tasks place greater cognitive demands, given that a “reorganization” of items is needed during retrieval.

The short-term memory processes used in the digit span forward test are also simpler than those deployed in performing the short and long word repetition test, which demand, besides participation of the phonoarticulatory loop and the central executive,^19^ involvement of long-term memory.^5^ The results, which revealed a similar difficulty in short and long word repetition, can be explained by the choice of words used in the test. The literature affirms that the performance of individuals in the repetition of short words is higher than in long words: the word length effect. Memorizing of short items is facilitated by the small space taken up in the WM by the information, allowing more items to be stored.^1,6.9,17.28-31^ The test we used contained words with two and three syllables considered short and long words, respectively. This difference of only one syllable may have led to the absence of the length effect. Studies investigating this effect^1,6,32^ used monosyllabic words as the short length form. Howard et al. demonstrated the length effect by comparing span between one, three and five syllable words. However, in the literature consulted, we found the experiment by Unswoth and Engle^30^ which verified the length effect using span between one and two syllable words. In the present study, which used two and three syllable words, this effect was not observed. However, as cited earlier, this effect may also have been influenced by the phonological complexity of the word and not only by its length.^32^ Moreover, word frequency was not controlled in the present study. All these aspects together might have interfered in the results found in the present study.

All WM tasks in the present study required a spoken response. Thus, the study was unable to disentangle WM per se from possible difficulties in speech production due to apraxia. Therefore, tasks in which WM is tested based on a response in a different modality would be necessary to confirm our hypothesis.

Furthermore, future studies should be carried out to elucidate the correlation between the degree of WM deficit and the degree of AOS. This would be valuable since the present study observed that all patients with AOS, independently of the severity of oral apraxia, presented some deficits in WM. Therefore, future studies should verify the relationship between the severity of apraxia and the severity of WM deficits.

In conclusion, evidence pointing to a lack of correlation between the degree of WM deficit presented by apraxics and the degree of apraxia of their speech was found in the present study.
